# Blood pressure level increase with altitude in three argentinean indigenous communities

**DOI:** 10.3934/publichealth.2019.4.370

**Published:** 2019-10-12

**Authors:** Valeria Hirschler, Claudio Gonzalez, Claudia Molinari, Hernan Velez, Mariela Nordera, Rodrigo Suarez, Alberto Robredo

**Affiliations:** 1 University of Buenos Aires, Buenos Aires, Argentina; 2 Cardiology, Hospital Materno Infantil, Salta, Argentina; 3 Hospital Oniativia, Salta, Argentina

**Keywords:** school children, high altitude, blood pressure

## Abstract

**Objective:**

To compare blood pressure (BP) levels in three groups of Argentinean Indigenous schoolchildren from similar ethnic backgrounds but living at three different altitudes.

**Methods:**

A cross-sectional study compared 185 (83 females) children aged 5–14 years from San Antonio de los Cobres (SAC), 3750 m above sea level; 46 (23 females) from Cobres, 3450 m; and 167 (83 females) from Chicoana (CH), 1400 m. Anthropometric and BP measurements were performed.

**Results:**

The prevalence of overweight/obesity was lower in SAC (6.5% [12]) and Cobres (4.3% [2]) than in CH (24% [24]) (BMI > 85 percentile per CDC norms). Systolic BP increased significantly with altitude: (SAC 86 mm Hg, Cobres 77 mm Hg, and CH 69 mm Hg). Similar results were obtained with diastolic BP (SAC 57 mm Hg, Cobres 51 mm Hg, and CH 47 mm Hg) and with median arterial pressure (MAP) (SAC 67 mm Hg, Cobres 60 mm Hg, and CH 55 mm Hg). Multiple linear regression analyses showed that altitude was significantly and independently associated with children's systolic BP (beta 10.56; R^2^ = 0.40), diastolic BP (beta 6.27; R^2^ = 0.25) and MAP (beta 7.69; R^2^ = 0.32); adjusted for age, sex, and BMI.

**Conclusions:**

We found that as altitude increased, BP levels increased significantly in indigenous children from similar backgrounds living permanently at different altitudes.

## 1. Introduction

Hypertension is the most important contributor to cardiovascular morbidity and mortality worldwide [1]. Few studies exist on blood pressure (BP) levels in high-altitude children living at an altitude of at least 3500 m [2]. Hypoxia due to high altitude exposes inhabitants to a low atmospheric pressure and is often combined with other factors such as cold weather, desert areas, limited diets, and poor socioeconomic conditions [3]. On the other hand, the main physiological adaptations that occur in individuals located at high altitudes are probably due to hypoxia [3]. Approximately 35 million people live in the Andean region in South America [4]. Contradicting results have been found in studies of the relationship between BP and chronic hypoxic-hypobaric conditions in individuals living at high altitude. One study performed in Nepalese individuals living at high altitude in a rural location found a lower prevalence of hypertension, suggesting that BP decreased with the increase in altitude [5]. However, previous research performed by our group showed that BP levels were significantly higher in children living at 3750 m compared with children from a community of a similar background but living at a lower altitude (1400 m) [6]. Therefore, it is important to assess the mechanisms and relationships between high altitude, and BP that play a role in determining health status at high elevations. As far as we know, there are no previous studies comparing three indigenous communities living at different altitudes in Andean northwestern Argentina. The objective of this study was to compare BP levels in three groups of Argentinean indigenous schoolchildren from similar ethnic backgrounds but living at three different altitudes.

## 2. Methods

### 2.1. Study design

This cross-sectional study was performed to compare BP levels between three indigenous communities living at different altitudes. The study was approved by the Human Rights Committee of the University of Buenos Aires. Each caregiver and child provided written informed consent after an explanation of the study and before its initiation.

This study included three indigenous communities of Diaguita descent in Salta province, northwestern Argentina. The sample size was calculated based on previous estimations, assuming an effect size for a one-way ANOVA of 0.50 with an alpha error of 0.05 and a power of 80% [6]. A minimum group size of 45 cases per altitude allowed us to detect a significant difference based on the previously assumed effect size [6].

Details of the San Antonio de los Cobres (SAC) and Chicoana (CH) communities have been previously reported [6]. Of the 3 schools located in SAC, one was chosen by simple randomization. Another school of comparable socioeconomic status was selected among the elementary schools located in CH. In each school, children were selected by simple randomization according to age group. The overall individual response rate was 87%. The third town, Cobres, is located in the Poma region at 3500 m above sea level, with a population of 141 inhabitants [7]. Since the total Cobres population is 141, the sample size of 47 elementary schoolchildren represents almost the whole population for this age group. All children from the only existing elementary school in Cobres were examined. The overall individual response rate was 94%. Exclusion criteria were as follows:

(1) Missing anthropometric measurements or blood pressure.

(2) The presence of a chronic disease or the use of medication that would affect blood pressure.

(3) Children that did not agree to participate.

Participants included in our study sample had no significant difference in socioeconomic level, age, BMI, and waist circumference, with those who were excluded because of missing data.

### 2.2. Data collection

Demographic data and children's anthropometry, BP, and lipid levels were assessed. Socioeconomic class included level of education and the presence or absence of a refrigerator or a dirt floor in the house. These two indicators are used by the National Statistics and Censuses Institute of Argentina to identify families of very low socioeconomic status [7]. Height and weight were measured with participants wearing light clothing, that is, without shoes, sweaters, or jacket. Body mass index (BMI) was calculated as weight (in kilograms) divided by height (in meters) squared. BP was measured by certified medical professionals in a standardized manner. Mercury sphygmomanometer measurements were performed by using a portable Baumometer sphygmomanometer Kompak Model-260 mm Hg (WA Baum, Copiague, NY). BP was measured with the child's right forearm resting horizontally on a table. After the appropriately sized cuff for each child was applied (covering approximately two-thirds of the arm), the cuff was slowly inflated to 20 mm Hg higher than the level at which the radial pulse receded. The commencement of sound (Korotkoff phase I) indicated systolic BP, and the disappearance of sound (Korotkoff phase V) indicated diastolic BP [8]. In the period of 1 to 2 minutes, two measurements were documented. The cuff was completely deflated between measurements. In the final analysis, the mean of the two measurements was calculated. Mean arterial pressure (MAP) is considered a better indicator of perfusion to vital organs than systolic BP. MAP was calculated using the following formula: diastolic BP multiplied by two plus systolic BP, divided by three. The MAP value was used because it constitutes a synthetic variable that integrates the values of both systolic and diastolic BP.

### 2.3. Definitions of overweight/obesity in children

BMI z-scores (BMI-z) were also determined [9]. Children were classified as underweight (< 5th percentile), normal weight (5th to < 85th percentile), overweight (85th to < 95th percentile), or obese (≥ 95th percentile) according to US Centers for Disease Control and Prevention (CDC) norms [9].

Blood samples were obtained from subjects after a 10 h overnight fast. All samples were analyzed in a single laboratory. We had stored SAC and CH serum samples at −70°C and both groups were assessed together. Serum lipids were measured using the Architect c16000 instrument (Toshiba, Kanagawa, Japan). Abnormal lipid levels were defined according to the National Institute of Health's Expert Panel on Integrated Guidelines for Cardiovascular Health and Risk Reduction in Children and Adolescents.

### 2.4. Statistical analysis

Descriptive statistics for raw variables are presented as mean ± SD values. An asymptotic Gaussian test was used to compare proportions due to the sample size. Variables with a skewed distribution were logarithmically transformed for analysis. Bonferroni's adjustment was carried out when many comparisons were performed. For the ANOVA test, homogeneity of variances was tested. When it was not validated, a Brown Forsythe test was performed.

The primary focus of the study was to compare BP levels between three indigenous communities living at different altitudes. Multiple linear regression analyses were performed to determine the association between BP levels and altitude adjusted for confounding variables. *P* values of < 0.05 were considered statistically significant. Analyses were performed using the IBM SPSS version 22.0 (IBM Corp., Armonk, NY, USA) statistical software package.

## 3. Results

### 3.1. General characteristics

**Figure 1.**
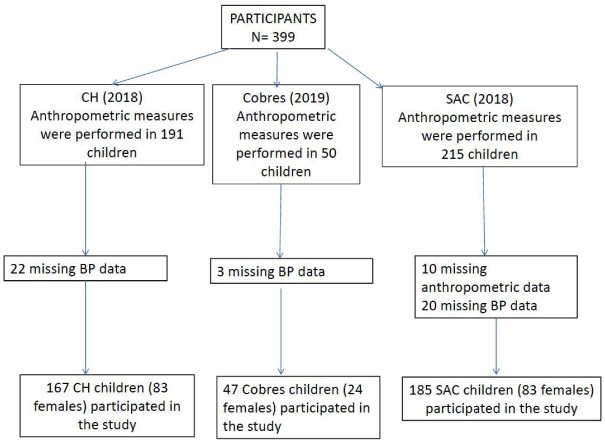
Study flowchart.

Anthropometric measurements were performed in 399 children. The study flowchart is shown in Figure 1. One hundred eighty-five schoolchildren (83 females) from SAC, 47 (24 females) from Cobres, and 167 children (83 females) from CH, aged 5–14 years, were included in the study. Forty-three percent of parents in SAC, 45% in Cobres, and 53% in CH had an elementary education or less; and 14.5% families in SAC, 22% in Cobres, and 2.8% in CH did not have a refrigerator at home or had a dirt floor (*P* < 0.01). Socioeconomic levels were significantly lower in Cobres and SAC than in CH, indicating that children living at higher altitudes belong to a lower socioeconomic class.

The prevalence of overweight/obesity was significantly lower in SAC (6.5% [12]) and Cobres (4.3% [2]) than in CH (24% [24]) (BMI > 85 percentile per CDC norms). There was not a significant difference in the prevalence of overweight/obesity according to sex in the 3 communities. In addition, there were no significant differences in waist circumference, BMI, BP, or lipid levels between sexes, except for diastolic BP (60 VS 5 mm gH) and MAP (69 VS 65 mm gH) in SAC children, which were significantly higher in girls than in boys. It is interesting to note that BP levels were not significantly different in children with overweight/obesity compared with children of normal weight in SAC and Cobres. However, in CH, overweight/obese children had significantly higher systolic BP (75 VS 67 mm Hg, respectively) and diastolic BP (51 VS 46 mm Hg, respectively) levels compared with normal weight children. This could be due to the lower prevalence of overweight/obesity in children living at very high altitudes (SAC and Cobres) compared with those living at 1400 m (CH).

Table 1 describes the clinical and metabolic characteristics of the sample. There were significant differences in mean age, waist circumference, BMI, BP, hematocrit, and lipid levels between CH, SAC, and Cobres children. Children who live at 3500 m (Cobres) had significantly lower oxygen saturation than those who live at 1400 m (CH). Unfortunately, we could not measure oxygen saturation in SAC. In general, children living at altitudes greater than or equal to 3500 m had significantly lower BMI, higher systolic and mean arterial pressure (MAP), higher hematocrit, and higher TG/HDL-C ratio. However, HDL-C levels were significantly lower in Cobres than in SAC and CH. As altitude increased, BMI adjusted for age and sex (z-BMI) increased significantly (Table 1). Furthermore, as altitude increased, systolic, and MAP increased significantly (Table 1).

**Table 1.** Anthropometric measurements, BP and lipid levels of children from SAC, Cobres and CH.

	CH (n = 167)	Cobres (n = 47)	SAC (n = 185)
Age (years)** ^(b)^	9.31± 2.12	8.17 ± 2.58	8.4 ± 2.39
Weight (Kg)**^(b)^	37.14± 13.79	26.52 ± 7.6	27.47 ± 9.04
Height (m)**^(b)^	1.37± 0.13	1.27 ± 0.15	1.27 ± 0.14
Waist (cm)**^(b)^	67.49 ± 12.75	56.51 ± 4.82	59.17 ± 8 .13
BMI**^(b)^	19.21 ± 4.46	16.09 ± 1.43	16.59 ± 2.62
BMI percentile**^(b)^	60.01 ± 28.69	44.11 ± 22.66	37.31 ± 26.15
z-BMI**^(b)^	0.37 ± 1.06	−0.15 ± 0.73	−0.43 ± 0.88
Systolic BP (mm Hg)**^(a)^	69.52 ± 14.39	77.54 ± 9.63	86.31 ± 12.97
Diastolic BP (mm Hg)**^(c)^	47.48 ± 10.9	51.52 ± 6.31	56.86 ± 12.78
MAP (mm Hg)**^(a)^	54.83 ± 11.62	60.2 ± 7.11	66.68 ± 12.32
Hematocrit (%)**^(b)^	41.10 ± 2.59	46.34 ± 2.32	44.25 ± 2.38
Oxygen Saturation (%)**^(a)^	95.42 ± 2.84	89.32 ± 7.47	
TG (mg/dL)	87.81 ± 41.21	96.47 ± 41.52	103.61 ± 39.13
HDL-C(mg/dL)** ^(e)^	48.4 ± 10.64	34.35 ± 8.59	45.77 ± 8.48
TG/HDL-C**^(a)^	1.97 ± 1.23	3.11 ± 1.86	2.39 ± 1.15

Note: SAC, San Antonio de los Cobres; CH, Chicoana; BMI, body mass index; Data were presented as mean ± SD. Z-score is a quantitative measure of the deviation of a specific variable taken from the mean of that population. Significance: **p<0.01. All means were significantly different. CH was significantly different than Cobres and SAC. SAC was significantly different than CH and Cobres. SAC was significantly different than CH. Cobres was significantly different than CH and SAC.

### 3.2. Correlations

Multiple linear regression analyses showed that altitude was significantly and independently associated with children's systolic BP (Table 2), diastolic BP (Table 3) and MAP (Table 4); adjusted for age, sex, and BMI. In addition, TG/HDL-C (beta 3.14; R^2^ = 0.1) and TG (beta 12.2; R^2^ = 0.12) were significantly and independently associated with altitude adjusted for age, sex, and BMI. When socio-economic variables were introduced as independent variables results did not change.

**Table 2.** Multiple regression analysis.

	Model	Non-Stand. Coefficients	Stand. Coefficients			
	B	Standard Error	Beta	t	Sig.	R^2^
Age	1.13	0.28	0.17	4.11	< 0.01	0.40
Sex	0.02	1.20	0.00	0.01	0.99	
Altitude	10.55	0.68	0.65	15.59	< 0.01	
BMI	1.18	0.18	0.28	6.48	< 0.01	
Dependent Variable SBP						

**Table 3.** Multiple regression analysis.

	Model	Non-Stand. Coefficients	Stand. Coefficients			
	B	Standard Error	Beta	t	Sig.	R^2^
Age	0.83	0.25	0.16	3.37	< 0.01	0.25
Sex	1.68	1.07	0.07	1.57	0.12	
Altitude	6.27	0.61	0.48	10.34	< 0.01	
BMI	0.87	0.16	0.26	5.29	< 0.01	
Dependent Variable DBP						

**Table 4.** Multiple regression analysis.

	Model	Non-Stand. Coefficients	Stand. Coefficients			
	B	Standard Error	Beta	t	Sig.	R^2^
Age	0.93	0.25	0.17	3.81	< 0.01	0.32
Sex	1.13	1.06	0.04	1.06	0.29	
Altitude	7.69	0.60	0.57	12.82	< 0.01	
BMI	0.97	0.16	0.28	6.00	< 0.01	
Dependent Variable MAP						

## 4. Discussion

We found that indigenous children living at 3500 m or higher above sea level had significantly higher BP levels than indigenous children living at 1,400 m. Furthermore, children living at high altitudes had a worse lipid profile than those living at 1400 m. A previous study performed by our group showed that SAC children had higher BP levels than CH [6]; however, there was no available information about BP levels in other communities living at 3500 m or higher. We found that the same phenomenon occurred in Cobres, a small community located in the Andean Mountains 70 km north of SAC.

It is important to note that most of these communities living at high altitude belong to a low socioeconomic class and struggle with a cold climate and limited access to urban centers [6]. One of the leading causes of stroke and myocardial infarction is high BP levels [10]. This could be due to several factors. One study found that the rate of hypertension in Tibetans living at 3000 m was 25% compared with 15% in non-Tibetans. The disparities in rates of hypertension between Tibetan and non-Tibetan high-altitude communities could be due to genetic or lifestyle behaviors. Tibetan diets consisted in high sodium consumption and few fruits and vegetables [11]. Therefore, the high prevalence of hypertension in Tibetans was attributable to an increased level of daily dietary salt intake [12]. Furthermore, Tibetans consumed a traditional salty tea which increased the salt intake by approximately four times the amount of salt recommended by the WHO [13]. The present study was performed in three communities with similar backgrounds. Although we did not measure the salt consumption in these Andean populations, these communities do not consume any kind of high sodium infusions. However, children living at 3500 m or higher had significantly higher BP levels compared with those from a similar ethnicity living at 1400 m. Other possible mechanisms involved in the higher rates of elevated BP in these high-altitude communities are cold temperatures and low socioeconomic levels. Higher exposure to cold weather and low socioeconomic status were associated with increased BP levels [14]. Consistently, the present study which compared two indigenous, low socioeconomic, and high-altitude communities showed that children living at 3500 m or higher in a windy and colder climate (mean temperature 45 °F) had higher BP levels than those living at 1400 m (mean temperature 63 °F). Biological adaptation to cold weather could have increased BP levels in these high-altitude communities.

A recent systematic review reported a prevalence of hypertension ranging from 23% to 56% in high-altitude populations of Tibet [15]. However, conflicting results have been gathered regarding the influence of altitude on BP. A study performed in an Andean population showed that systolic BP was lower in 3 Peruvian communities located at 4000 m compared with two communities at sea level [2]. The authors suggested that the lower BP levels could be associated with increasing intake of zinc and healthier diets [2]. Furthermore, a Peruvian study that compared women from Quechua origins living at 3900 m with those living in Lima showed that fat mass and diastolic BP were significantly lower in women living at high altitudes [16]. The authors suggested that this could be due to the fact that women living at 3800 m in Cuzco walked more than 1 hour per day up and down hills while women from Lima had a more sedentary lifestyle, even though their diets were similar. In addition, studies based on populations from South America showed low BP values at high altitude [16]–[18]. However, a study conducted in Lhasa, the capital city of Tibet, located at 4300 m above sea level, showed that the age-standardized prevalence of hypertension was 57.1% in adults aged 40 years or older [11]. Furthermore, another study conducted in Lhasa in individuals aged 18 years or older showed that approximately 40% had hypertension [19]. In addition, exposure to 3400 m and 5400 m above sea level was associated with a progressive increase in either conventional or ambulatory SBP and DBP [20]. Consistently, we found that Andean children from 2 communities living at 3500 m or higher had significantly higher systolic BP and MAP levels than children living at 1400 m. A cross-sectional study performed in 370 Tibetans living at 3660 m above sea level in Lhasa found high rates of dyslipidemia, another cardiovascular risk factor [21]. Accordingly, the present study showed higher triglycerides and triglycerides/HDL-C levels in children living at 3500 m or higher.

It is interesting to note that the 3 communities studied belonged to similar ethnic backgrounds. Different mechanisms had been proposed. Chronic hypoxic-hypobaric conditions in individuals living at high altitude could be associated with an increase cardiovascular risk [3]. A study showed that noradrenaline increased whereas angiotensin II and aldosterone decreased with the exposure to an altitude of 3400 m and 5400 m [20]. Consistently, we found that children from Cobres (3500 m) had a lower oxygen saturation than children living in CH (1400 m); suggesting that higher altitude are associated with lower oxygen saturation and increased cardiovascular risk. Consistently, an additional study measured sympathetic nerve activity using peroneal microneurography in 8 individuals aged 24 years who were exposed to an altitude of 5260 m in the Bolivian Andes mountains for 4 weeks. They found that sympathetic nerve activity was significantly higher at 5260 m compared with sea level [22]; suggesting that an increase in sympathetic activity was associated with higher BP levels in individuals living at high altitude. Another possible mechanism involved in the higher BP levels was an increase of blood viscosity because of an increase in hematocrit in individuals exposed to high-altitude [20]. Consistently, a previous study performed by our group found that SAC children had significantly higher hemoglobin levels than children living at sea level [23]. Accordingly, this study shows that children living at 3500 m or more had significantly higher hematocrit than those living at 1400 m. Another possible mechanism is related to the decrease in partial pressure gradients that interfere with the exchange of gas, causing chronic insufficiency of oxygenated blood circulation. Therefore, decreasing the supply of oxygenated blood to the kidneys leads to the release of renin, which contributes to the vasoconstriction of arteries and the subsequent increase in BP [24]. However, the underlying mechanisms leading to elevated BP in individuals exposed to hypobaric hypoxia has not yet been elucidated. To our knowledge, this is the first study showing higher BP levels among Indigenous children living at altitudes greater than 3500 m compared with Indigenous children living at 1400 m.

Study limitations: First, we could not determine a causal relationship between BP levels and altitude because of the cross-sectional study design. Second, because of economic limitations, ambulatory BP monitoring over 24 h was not performed, even though the effects of hypoxia are more evident in 24 h ambulatory BP monitoring than in conventional BP readings [20]. Third, salt intake was not measured in these communities. Fourth, even though children from these communities have little mixture with other populations, we do not have hard data (genetic markers) to confirm these findings. Fifth, unfortunately, due to budget limitations we could not measure oxygen saturation in SAC children. However, oxygen saturation was measured in children from Cobres (3500 m) and CH (1400 m). Finally, no control group at sea level was included; therefore, while we could compare 3 different altitudes, we could not know whether subjects living at 1400 m could be considered similar or not to those living at sea level.

Despite these limitations, the strengths of this study include the similarities in the ethnic backgrounds among children from these communities living at 3 different altitudes, which allowed us to examine correlations independently of ethnicity. Furthermore, there was a high response rate of the children, and the study was performed in each child through direct measurements taken by our team rather than self-reported data. Finally, we used regression models and simultaneous adjustment of confounding variables.

Conclusions: Children living at 3750 m and 3500 m above sea level had higher BP levels than those living at 1400 m. Given the long lag time between the detection of cardiovascular risk factors and the onset of clinical cardiovascular disease, screening of blood pressure levels might provide a unique opportunity for intervention in these communities. Future longitudinal studies should be performed to confirm these findings.

## References

[1] Forouzanfar MH, Alexander L, Anderson HR (2015). GBD 2013 Risk Factors Collaborators. Global, regional, and national comparative risk assessment of 79 behavioural, environmental and occupational, and metabolic risks or clusters of risks in 188 countries, 1990–2013: a systematic analysis for the Global Burden of Disease Study 2013. Lancet.

[2] Ruiz L, Peñaloza D (1977). Altitude and hypertension. Mayo Clin Proc.

[3] Miele CH, Schwartz AR, Gilman RH (2016). Increased Cardiometabolic Risk and Worsening Hypoxemia at High Altitude. High Alt Med Biol.

[4] Moore LG, Niermeyer S, Zamudio S (1998). Human adaptation to high altitude: regional and life-cycle perspectives. Am J Phys Anthropol.

[5] Shrestha S, Shrestha A, Bhattarai D (2012). Blood pressure in inhabitants of high altitude of Western Nepal. J Nepal Med Assoc.

[6] Hirschler V, Molinari C, Maccallini G (2019). Blood Pressure Levels Among Indigenous Children Living at Different Altitudes. Appl Physiol Nutr Metab.

[7] INDEC (2010). Instituto Nacional de Estadística y Censos de la República Argentina. http://www.censo2010.indec.gov.ar/<zurlx>.

[8] Flynn JT, Kaelber DC, Baker-Smith CM (2017). Clinical practice guideline for screening and management of high blood pressure in children and adolescents. Pediatrics.

[9] Kuczmarski RJ, Ogden CL, Guo SS (2002). 2000 CDC growth charts for the United States: methods and development. Vital Health Stat.

[10] Lim SS, Vos T, Flaxman AD (2012). A comparative risk assessment of burden of disease and injury attributable to 67 risk factors and risk factor clusters in 21 regions, 1990–2010: a systematic analysis for the Global Burden of Disease Study 2010. Lancet.

[11] Zhao X, Li S, Ba S (2012). Prevalence, awareness, treatment, and control of hypertension among herdsmen living at 4300 m in Tibet. Am J Hypertens.

[12] Tian HG, Guo ZY, Hu G (1995). Changes in sodium intake and blood pressure in a community-based intervention project in China. J Hum Hypertens.

[13] Liu L, Ding Y, Huang Z (2001). Ethnic and environmental differences in various markers of dietary intake and blood pressure among Chinese Han and three other minority peoples of China: results from the WHO Cardiovascular Diseases and Alimentary Comparison (CARDIAC) Study. Hypertens Res.

[14] Mitchell R, Blane D, Bartley M (2002). Elevated risk of high blood pressure: climate and the inverse housing law. Int J Epidemiol.

[15] Mingji C, Onakpoya IJ, Perera R (2015). Relationship between altitude and the prevalence of hypertension in Tibet: a systematic review. Heart.

[16] Lindgärde F, Ercilla MB, Correa LR (2004). Body adiposity, insulin, and leptin in subgroups of Peruvian Amerindians. High Alt Med Biol.

[17] Makela M, Barton SA, Schull WJ (1978). The Multinational Andean Genetic and Health Program—IV. Altitude and the blood pressure of the Aymara. J Chronic Dis.

[18] Hernandez-Hernandez R, Silva H, Velasco M (2010). Hypertension in seven Latin American cities: the cardiovascular risk factor multiple evaluation in Latin America (CARMELA) study. J Hypertens.

[19] Chen W, Liu Q, Wang H (2011). Prevalence and risk factors of chronic kidney disease: a population study in the Tibetan population. Nephrol Dial Transplant.

[20] Parati G, Bilo G, Faini A (2014). Changes in 24h ambulatory blood pressure and effects of angiotensin II receptor blockade during acute and prolonged high-altitude exposure: a randomized clinical trial. Eur Heart J.

[21] Sherpa LY, Stigum H, Chongsuvivatwong V (2011). Lipid profile and its association with risk factors for coronary heart disease in the highlanders of Lhasa, Tibet. High Alt Med Biol.

[22] Hansen J, Sander M (2003). Sympathetic neural overactivity in healthy humans after prolonged exposure to hypobaric hypoxia. J Physiol.

[23] Hirschler V, Maccallini G, Aranda C (2012). Dyslipidemia without Obesity in Indigenous Argentinean Children Living at High Altitude. J Pediatr.

[24] Howden R, Kleeberger SR (2012). Genetic and environmental influences on gas exchange. Compr Physiol.

